# Supplementary material for: Transfer of Cardiac Mitochondria Improves the Therapeutic Efficacy of Mesenchymal Stem Cells in a Preclinical Model of Ischemic Heart Disease
Source: Cells. 2023 Feb 11;12(4):582. doi: 10.3390/cells12040582 (PMC9953768; doi:10.3390/cells12040582)
Supplement: Supplementary file 1 [file cells-12-00582-s001.zip › cells-2135007-SI.pdf]

cells

MDPI

Supplementary Materials

Transfer of Cardiac Mitochondria Improves the Therapeutic Efficacy of Mesenchymal Stem Cells in a Preclinical Model of Ischemic Heart Disease

Marie-Luce Vignais [1], Jennyfer Levoux [2,3], Pierre Sicard [4], Khattar Khattar [1], Catherine Lozza [4], Marianne Gervais [2], Safia Mezhoud [2], Jean Nakhle [1], Frederic Relaix [2,5,6], Onnik Agbulut [3], Jeremy Fauconnier [4] and Anne-Marie Rodriguez [2,3,6]*

[1] IGF, Univ. Montpellier, CNRS, INSERM, 34094 Montpellier, France; marie-luce.vignais@igf.cnrs.fr (M.-L.V.); khattar.khattar@igf.cnrs.fr (K.K.); jean.nakhle@inserm.fr (J.N.)
[2] Université Paris-Est Créteil, INSERM, IMRB, 94010 Créteil, France; jennyfer.levoux@inserm.fr (J.L.); taurel@u-pec.fr (M.G.); safia.mezhoud@inserm.fr (S.M.); frederic.relaix@inserm.fr (F.R.)
[3] Sorbonne Université, Institut de Biologie Paris-Seine (IBPS), CNRS UMR 8256, INSERM U1164, Biological Adaptation and Ageing, 75005 Paris, France; onnik.agbulut@sorbonne-universite.fr
[4] PhyMedExp, Inserm, CNRS, University of Montpellier, 34295 Montpellier, France; pierre.sicard@inserm.fr (P.S.); catherine.lozza@umontpellier.fr (C.L.); jeremy.fauconnier@inserm.fr (J.F.)
[5] École Nationale Vétérinaire d'Alfort, IMRB, 94700 Maisons-Alfort, France
[6] APHP, Hôpitaux Universitaires Henri Mondor & Centre de Référence des Maladies Neuromusculaires GNMH, 94000 Créteil, France
* Correspondence: anne-marie.rodriguez@inserm.f

Table S1. Human and mouse primer sequences.

Gene	Human primer sequences	Gene	Mouse primer sequences
TBP	Forward : 5'-AACAACAGCCTGCCACCTTA-3‘<br>Reverse : 5'-GGGTCAGTCCAGTGCCATAA-3'	***TBP***	Forward: 5'- ATCCCAAGCGATTTGCTG -3‘<br>Reverse: 5'- CCTGTGCACACCATTTTTCC -3'
Ki67	Forward: 5’-ACGTCGTGTCTCAAGATC-3’<br>Reverse: 5’-CGGTACTGTCTTCTTTGAC-3’	***VEGF***	Forward: 5'-AACGATGAAGCCCTGGAGTG-3‘<br>Reverse: 5'-TGAGAGGTCTGGTTCCCGA-3'
VEGF	Forward: 5'-AGAAGGAGGAGGGCAGAATC-3‘<br>Reverse: 5'-ACACAGGATGGCTTGAAGATG-3'	***VEcadherin***	Forward: 5'-TTGGGCTTTCTGACTGTTGT-3‘<br>Reverse: 5'-CAGGGACTTCGTGGGTTT-3'
HGF	Forward: 5'-CGACAGTGTTTCCCTTCTCG-3‘<br>Reverse: 5’-AAGGCTTCAAGGCATCTG-3’	***CD31***	Forward: 5'-CGGTTATGATGATGTTTCTGGA-3‘<br>Reverse: 5'-AAGGGGAGGACACTTCCACTTCT-3'
CXCL1	Forward: 5’-TAAAGGTAGCCCTTGTTTCCCC-3’<br>Reverse: 5’-CAGACCACGCAAGGAGTTCA-3’	***CD206***	Forward: 5’-CTCTGTTCAGCTATTGGACGC-3’<br>Reverse: 5’-CGGAATTTCTGGGATTCAGCTTC-3’
CXCL5	Forward: 5’-CTTCCACCTTGGAGCACTGT-3’<br>Reverse: 5’-CCAAGGTGGAAGTGGTAGCC-3’	***Arg1***	Forward: 5’-CTCCAAGCCAAAGTCCTTAGAG-3’<br>Reverse: 5’-AGGAGCTGTCATTAGGGACATC-3’
CXCL6	Forward: 5’-TTTAGAAAAGGGGCTTCCGGG-3’<br>Reverse: 5’-GCGTTTGAGTCAGCAAAGAAGTC-3’	***CHIA***	Forward: 5’-ATGGCCAAGCTACTTCTCGTCACA-3’<br>Reverse: 5’-TCCCAGCAAAGGCATAGATCAGGT -3’
IL8	Forward: 5’-TTCCTTGGGGTCCAGACAGA-3’<br>Reverse: 5’-CATGAACTGTGTTTGCCGC-3’	***CHIL3***	Forward: 5’-TGCGTGACTATGAAGCATTGAAT-3’<br>Reverse: 5’-CCAATGGCCAGGAGAGTTTTT-3’
IL11	Forward: 5’-CAGGGAATCCAGGTTGTGGTC-3’<br>Reverse: 5’-GAAAATCCCAACAGAAGGCCAAA-3’	***iNOS***	Forward: 5'-CCACCAACAATGGCAACATCAGGT-3‘<br>Reverse: 5'-TAGGTCGATGCACAACTGGGTGAA-3'
IL33	Forward: 5’-TAACTCAGTAACACCTTATCTGTGA-3’<br>Reverse: 5’-CCAACGTGACGGACTTCCC-3’	***TN[illegible]a***	Forward: 5’-AATGGCCTCCCTCTCATCAGTT-3’<br>Reverse: 5’-CGAATTTTGAGAAGATGATCTGAGTGT-3’

LIF	Forward: 5’-TACACGACTATGCGGTACAGC-3’<br>Reverse: 5’-CTGGCGATACCTCAGCAACC-3’
MMP1	Forward: 5’-GGTGTGACATTACTCCAGAGTTG-3’<br>Reverse: 5’-GGACTTAGACCGCTTGGCTT-3’
MMP9	Forward: 5’-TTCAGGGCGAGGACCATAGA-3’<br>Reverse: 5’-TCCAGCAACTTTATGGGGGT-3’
MMP14	Forward: 5’-TTCCCGTCACAGATGTTGGG-3’<br>Reverse: 5’-TTCCCGTCACAGATGTTGGG-3’

LIF	Forward: 5’-GCTGTATCGGATGGTCGCATA-3’<br>Reverse: 5’-CACAGACGGCAAAGCACATT-3’
Co[illegible]-1	Forward: 5’-GACGCCATCAAGGTCTACTG-3’<br>Reverse; 5’-ACGGGAATCCATCGGTCA-3’
Co[illegible]-3	Forward: 5’-GAGGAATGGGTGGCTATCCG-3’<br>Reverse: 5’-GCGTCCATCAAAGCCTCTGT-3’
Fib[illegible]onectin-1	Forward: 5’-CCAGGCACTGACTACAAGAT-3’<br>Reverse: 5’-CATGATACCAGCAAGGAGT-3’
